# Wedge Resection vs. Stereotactic Body Radiation Therapy for Clinical Stage I Non-small Cell Lung Cancer: A Systematic Review and Meta-Analysis

**DOI:** 10.3389/fsurg.2022.850276

**Published:** 2022-03-17

**Authors:** Lei Peng, Han-Yu Deng, Zhen-Kun Liu, Qian-Wen Shang, Kai-Li Huang, Qiang-Qiang Zheng, Wen Li, Yun Wang

**Affiliations:** ^1^Lung Cancer Center, West China Hospital of Sichuan University, Chengdu, China; ^2^Department of Thoracic Surgery, West China Hospital of Sichuan University, Chengdu, China; ^3^Department of Clinical Lab, Chongqing University Cancer Hospital and Chongqing Cancer Hospital, Chongqing, China

**Keywords:** non-small cell lung cancer, clinical stage I, wedge resection, SBRT, prognosis

## Abstract

**Background:**

Whether wedge resection or stereotactic body radiation therapy (SBRT) has better effectiveness in treatment of clinical stage I non-small cell lung cancer (NSCLC) patients remains unclear. Here we conducted the first meta-analysis to directly compare the survival outcomes of clinical stage I NSCLCs treated with wedge resection and SBRT.

**Methods:**

We systematically searched studies from PubMed, Embase, and Corchrane Library up to October 1, 2021. Data for analysis mainly included overall survival (OS) and disease-free survival (DFS), which were obtained directly from the text results or calculated from the Kaplan–Meier survival curve. We used the standard random-effect model test (DerSimonian and Laird method) to analyze the pooled hazard ratios (HRs) and 95% confidence intervals (CIs). The *Q*-test and *I*^2^-test were used to assess heterogeneity. The stability of pooled HRs was examined by sensitivity analysis.

**Results:**

Six retrospective studies with a total of 11,813 clinical stage I NSCLCs who received wedge resection or SBRT were included. The results showed that patients receiving wedge resection had a significantly better OS (HR = 1.20, 95% CI = [1.07, 1.34], *P* = 0.002) than those with SBRT, but no significant difference of DFS (HR 1.53, 95% CI = [0.83–2.83], *P* = 0.17) was observed. There was no significant heterogeneity during our analysis, but there may be potential publication bias among these studies.

**Conclusions:**

Our meta-analysis showed that clinical stage I NSCLCs treated with wedge resection had superior OS than those treated with SBRT. However, more prospective clinical trials should be well-designed to evaluate the optimal treatment modality of early-stage NSCLCs.

## Introduction

Lung cancer causes the most cancer-related deaths worldwide (1). The two major types of lung cancer are non-small-cell lung cancer (NSCLC) and small cell lung cancer (SCLC), of which the former accounts for about 80% (2). Nowadays, with the routine use of computed tomography (CT), more and more early-stage NSCLCs are being detected (3). Surgery plays an important role in the treatment of early-stage NSCLC and it mainly consists of lobectomy, sleeve lobectomy, sublobar resection (segmentectomy and wedge resection) and so on. Among them, lobectomy with systematic lymph node dissection is still the golden standard of medically operable patients with clinical stage I NSCLC (4). However, when patients are unsuitable for lobectomy or with low grade malignancy peripheral-type NSCLC <2 *cm*, sublobar resection is recommended (5). Moreover, previous study (6) found that among elderly aged (≥75 years) stage I NSCLC patients, those treated with sublobar resection had similar OS compared with lobectomy, but yielded less postoperative complications, which suggested sublobar resection but not lobectomy may be the optimal treatment for elderly stage I NSCLC patients.

On the other hand, with the progress of medical treatment, stereotactic body radiation therapy (SBRT), also known as stereotactic ablative radiotherapy (SABR), is the standard treatment for medically inoperable early-stage NSCLC patients (7). Furthermore, several reports showed SBRT had a good local control in the medically inoperable patients, ranging from 80 to 99% (8–11). However, it's still controversial whether there is still a role of SBRT for operable early-stage NSCLC patients. Although a large number of retrospective observational studies directly compared surgery and SBRT for operable early-stage NSCLCs, they have reported conflicting results. It still has limited information to guide clinicians for decision-making because there have not been a completed prospective trial and several (POSITIVL, VALOR and STABLE-MATES) (12–14) randomized controlled trial (RCTs) are still ongoing.

In our previous meta-analysis (15), we made a comparison between SBRT and surgery in treating stage I NSCLC. In the subgroup analysis, we found that SBRT and sublobar resection yielded similar 3-year survival rate, OS and 3-year loco-regional control (LRC) rate, while lobectomy yielded significantly longer OS than SBRT. However, our sublobar resection subgroup included both wedge resection and segmentectomy, whether wedge resection or segmentectomy resulted in the similar efficacy to SBRT in patients with stage I NSCLC remains unclear. Besides, segmentectomy and wedge resection are different surgical procedures, and previous study showed segmentectomy was associated with better OS and cancer–specific survival (CSS) compared with wedge resection in treating NSCLC <2 cm (16). In our opinion, both wedge resection and SBRT are local therapies, provide better lung function preservation and can be well-tolerated by most of the patients (17). The comparison between wedge resection and SBRT may be more appropriate than other types of surgical resections. Hence, with a growing number of previous similar studies (18–23), we performed the first meta-analysis to investigate whether wedge resection or SBRT had a better effect in clinical stage I NSCLCs.

## Methods

Our meta-analysis was conducted in accordance to the preferred Reporting Items for Systematic Reviews and Meta-Analyses (PRISMA) guidelines (24). All the data were collected from published papers and ethical approval was waived from our hospital. The present meta-analysis was registered in the PROSPERO database.

### Search Strategy

We used the following searching strategy: (“lung carcinoma” OR “lung cancer” OR “lung neoplasms”) AND (“wedge ” OR “wedge resection” OR “sublobar resection”) AND (“stereotactic body radiotherapy” OR “SBRT” OR “Stereotactic body radiation therapy” OR “SABR” OR “Stereotactic ablative radiotherapy”) to search the electronic database of PubMed, Embase, and Cochrane Library from inception until October 1, 2021. Only papers published in English were evaluated.

### Inclusion Criteria and Exclusion Criteria

Before we searched eligible literatures to conduct this meta-analysis, the following inclusion criteria were set, (1) RCT or observational study, (2) comparison between stage I NSCLC patients treated with wedge resection and SBRT, (3) study provided sufficient survival data [OS or disease-free survival (DFS)] for analysis. We also made the following criteria for study exclusion: (1) study written in non-English language; (2) review, case report, conference abstract, and editorial material and letters; (3) study did not report OS or DFS.

### Data Extraction and Quality Assessment

Two authors (Peng and Huang) screened the titles and abstracts to find potentially available ones according to the inclusion and exclusion criteria. And another author (Deng) would solve the disagreement if there was. After browsing and evaluating the full text that met the inclusion criteria, the two authors independently extracted the following information: (1) language, author, publication date and study type; (2) patients' quantity of each group, median age, median follow-up time and radiation therapy strategy. If primary reports didn't directly record the data, we calculated HRs with 95% CIs with spreadsheet according to the survival curves with the published methodology by analyzing Kaplan–Meier curves of the included studies with Engauge Digitizer version 2.11. Subsequently, for observational studies, we used Newcastle–Ottawa Scale (NOS) (25) to evaluate the risk of bias, which contained three factors: (1) selection of study group, (2) comparability of groups, and (3) assessment of outcomes. Papers with a quality score >6 were considered high-quality. For RCTs, we used Jadad Scale (26) to assess the methodological quality, and if the score >4, the paper was defined as high-quality paper.

### Statistical Analysis

We performed a meta-analysis of wedge resection vs. SBRT and two subgroup meta-analyses (one meta-analysis of wedge resection vs. SBRT with propensity-matched (PSM) cohort and another without PSM cohort). We used the software Review Manager (RevMan) version 5.4 (the Cochrane Collaboration, Oxford, England) and STATA (StataCorp;College Station, TX, USA) 12.0 package to perform all statistical analysis based on the PRISMA guidelines (24). Quantitative synthesis of the 5-year OS and 5-year DFS was conducted by using HRs with 95% CIs, and it was considered high heterogeneity if an *I*^2^ ≥ 50% or *P* ≤ 0.10 was detected. When we encountered high heterogeneity, the random-effect model test (DerSimonian and Laird method) was performed for statistical analysis. Otherwise, the standard fixed-effect model test (Mantel-Haenszel method) was available. The sensitivity analysis was performed to estimate the stability of quantitative synthesis results, and the reliability of the meta-analysis results were robustly confirmed if the results of the meta-analysis did not change significantly. To evaluate publication bias, a funnel plot with Begger's and Egger's tests was used.

## Results

### Study Selection

**Figure 1:**
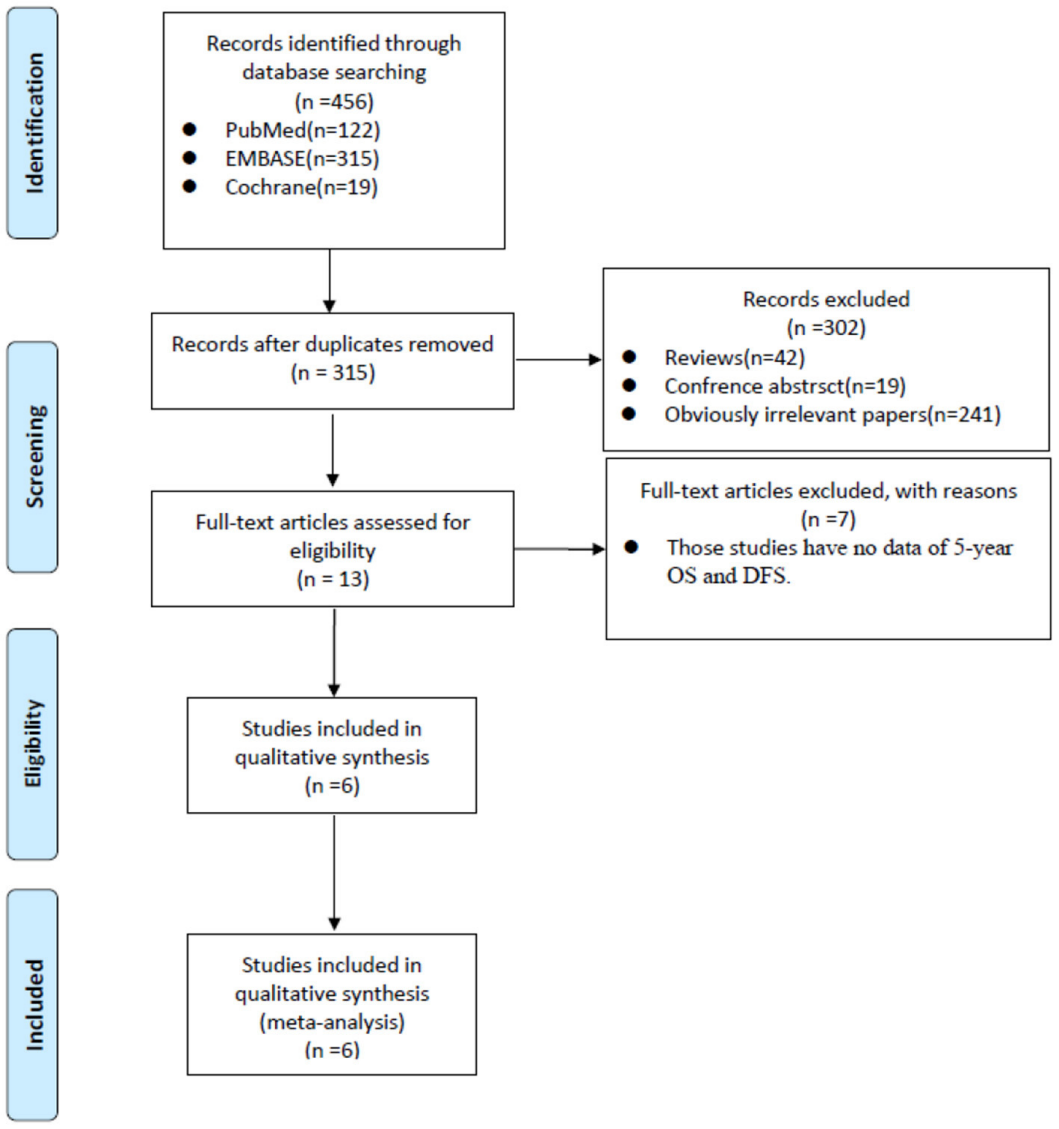
PRISMA flow diagram showed the process of relevant studies evaluation. PRISMA, Preferred Reporting Items for Systematic Reviews and Meta-analysis; OS, overall survival; DFS, disease-free survival.

A total of 456 papers were identified from the three main sources including Pubmed, Embase and Cochrane Library. After we deleted 141 duplicates. By browsing titles and abstracts, we excluded 42 reviews, 19 conference abstracts, and 241 obviously irrelevant papers. In the remaining 13 papers, we further excluded another seven papers by full-text assessment and finally, six studies (18–23) were included in the final meta-analysis (Figure 1).

### Characteristics of Eligible Studies

In summary, all of the six retrospective observational studies contained 11,813 clinical stage I NSCLC patients, and the publication year ranged from 2013 to 2020. One study did not report follow-up time, and the others' median follow-up time ranged from 17.5 to 66 months. Three studies did not report radiation dose and cycles, and the others' radiation dose ranged from 30–60, 3–5 cycles. The NOS scores ranged from 7 to 9, which meant our meta-analysis had a low risk of bias. Detailed characteristics of the included studies were shown in Table 1.

**Table 1:** Characteristics of selected studies.

**Study**	**Language**	**Period**	**Stage**	**Median age (years)**		**Radiation dose (Gy/no.)**	**Sample number**			**Median follow-up (months)**	**Study type**	**Quality assessment**
				**WR**	**SBRT**		**Total**	**WR**	**SBRT**			
Yerokun et al. (21)	English	2008–2011	IA	73	73	NR	3,168	1,584	1,584	NR	ROS	NOS:8
Parashar et al. (20)	English	1993–2012	I	77		30–60/3–5	220	123	97	17.5	ROS	NOS:7
Ajmani et al. (22)	English	2003–2015	I	73.9	73.8	NR	7,734	3,867	3,867	66	ROS	NOS:9
Varlotto et al. (18)	English	1999–2008	I	67.5	73.3	48–60/3-5	34	17	17	25.8	ROS	NOS:7
Mayne et al. (23)	English	2004–2015	IA	73	73	NA	558	279	279	27.6	ROS	NOS:8
Port et al. (19)	English	2001–2012	IA	72	76	30–60/3–5	99	76	23	35	ROS	NOS:7

*WR, wedge resection; SBRT, stereotactic body radiation therapy; NOS, Newcastle-Ottawa Scale; NR, not reported; ROS, retrospective observational studies*.

### Meta-Analysis of Overall Survival Analysis

**Figure 2:**
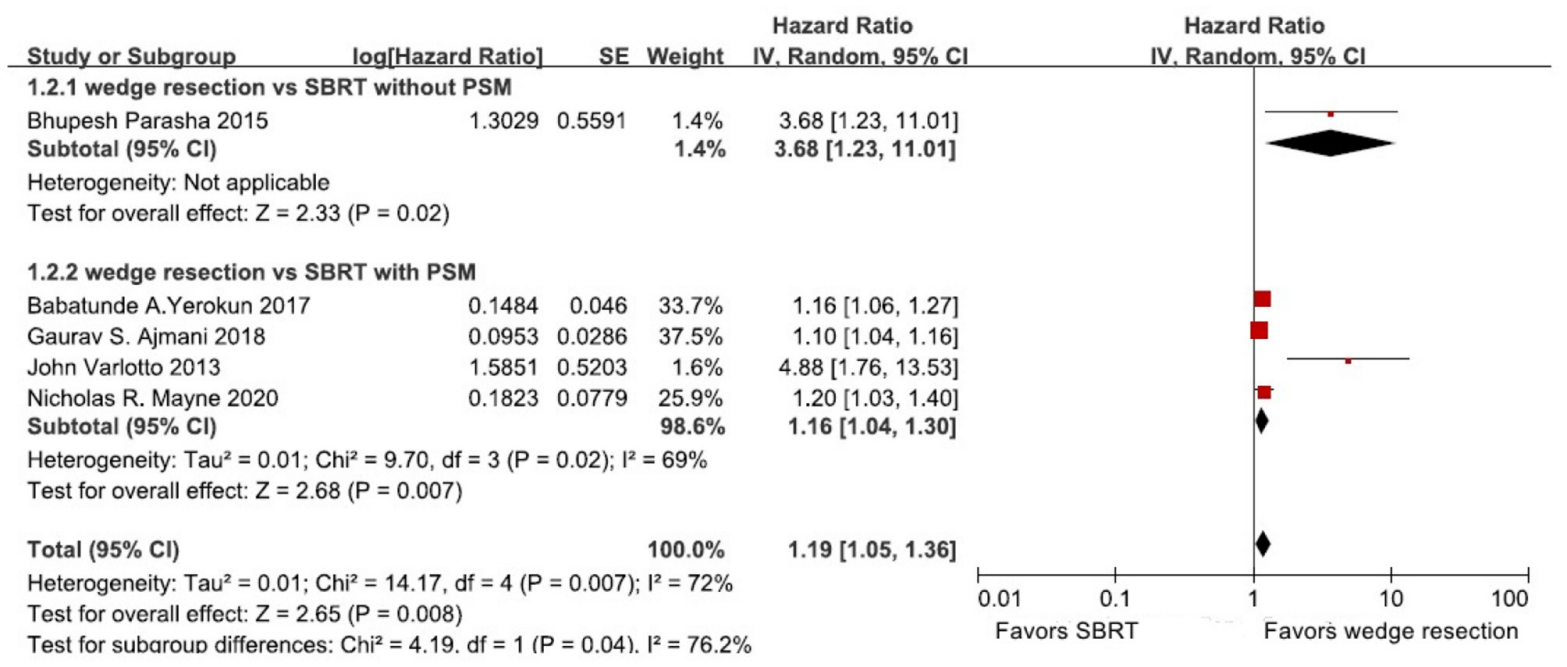
Forest plot of 5-year OS in patients treated with wedge resection compared with SBRT; OS, overall survival; CI, confidence interval; PSM: propensity score-matched.

Four studies directly reported 5-year OS rates of wedge resection vs. SBRT. One study showed the OS rate in a Kaplan-Meier survival curve. And therefore, Engauge Digitizer version 2.11 and HR calculation spreadsheet were used to calculate HRs and 95% CIs of OS. Five studies were included to calculate the HR and 95% CI of OS (Table 2). The heterogeneity test showed that there was high heterogeneity among these studies (*I*^2^ = 72%, *P* = 0.007). Therefore, we use the random-effects model to perform this meta-analysis, and the results showed that stage I NSCLC patients treated with wedge resection had a better OS compared to SBRT (HR = 1.19, 95% CI = [1.05, 1.36], *P* = 0.008) (Figure 2). In our subgroup analysis, four studies with propensity score-matched analysis including 11,494 patients were analyzed. We found that patients treated with wedge resection still yielded a significantly higher OS rate than SBRT (random-effects model: (HR = 1.16, 95% CI = [1.04, 1.30], *P* = 0.007, *I*^2^ = 69%) (Figure 2). Because only one study reported 5-year LCR rate, we did not perform further meta-analysis based on 5-year LRC rate.

**Table 2:** Main outcomes extracted from the selected studies.

**Study**	**5-year OS rate**		**5-year DFS rate**		**5-year LRC rate**		**OS**		**DFS**	
	**WR**	**SBRT**	**WR**	**SBRT**	**WR**	**SBRT**	**HR**	**95% CI**	**HR**	**95% CI**
Yerokun et al. (21)	49.9	31.0	NR	NR	NR	NR	1.16	1.06–1.27	NR	NR
Parashar et al. (20)	97.7	89.6	50.5	46.9	NR	NR	3.68	1.23–11.01	1.23	0.85–1.78
Ajmani et al. (22)	49.5	33.0	NR	NR	NR	NR	1.10	1.04–1.16	NR	NR
Varlotto et al. (18)	86.3	31.7	NR	NR	92.9	77.1	4.88	1.76–13.53	NR	NR
Mayne et al. (23)	53.0	31.0	NR	NR	NR	NR	1.20	1.03–1.40	NR	NR
Port et al. (19)	NR	NR	63.8	41.3	NR	NR	NR	NR	2.39	1.03–5.55

*WR, wedge resection; SBRT, stereotactic body radiation therapy; OS, overall survival; DFS, disease-free survival; LRC, loco-regional control; CI, confidence interval; HR, hazard ratio; NR, not reported*.

### Meta-Analysis of Disease-Free Survival Analysis

**Figure 3:**
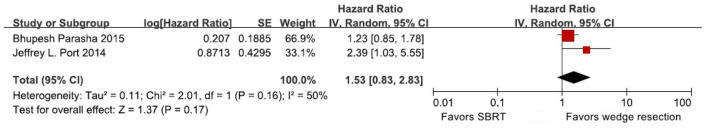
Forest plot of 5-year DFS in patients treated with wedge resection compared with SBRT. CI, confidence interval; DFS, disease-free survival.

One study provided the 5-year DFS rate of wedge resection vs. SBRT, and one study reported 3-year DFS rate, as it showed the DFS rate in a Kaplan-Meier survival curve. Therefore, Engauge Digitizer version 2.11 and HR calculation spreadsheet were used to calculate HRs and 95% CIs of DFS (Table 2). Because the heterogeneity was high (*I*^2^ = 50%, *P* = 0.16), and a random-effects model was used for analysis. The results indicated that there was no significant differences of DFS between patients treated with wedge resection and SBRT (HR 1.53, 95% CI = [0.83–2.83], *P* = 0.17) (Figure 3).

### Sensitivity Analysis and Publication Bias

We conducted the sensitivity analysis to assess the stability of our meta-analysis results. We sequentially removed each study, and the results showed the overall results of OS did not change, which meant our meta-analysis results had high stability (Supplementary Figure 1). However, when we performed a funnel plot to evaluate publication bias, it showed there was potential publication bias among the studies, as the appearance of funnel plot was asymmetry, and Begger's and Egger's tests of OS (*P* = 0.003, Supplementary Figure 2) was statistical significance.

## Discussion

The incidence of NSCLC is increasing and has become a global health problem (1). With the application of multidisciplinary treatment model in treating lung cancer, the 38survival rate of NSCLC patients has improved (27). Traditionally, surgery is the first choice of operable early-stage NSCLC, and lobectomy with systematic lymph node dissection has become the acceptable treatment of stage I NSCLC since 1962 (28). While about 20 to 25% of early-stage NSCLC patients are not the right candidates for lobar resection because of the poor physical conditions (27), some other patients also refuse surgery. For this part of patients, SBRT has been developed as an alternative treatment during the past few years (7, 29). However, whether surgery or SBRT plays an important role on the treating for early-stage NSCLC patients, still has no consensus since numerous non-randomized data have reported different results (30–33). The only two independent RCTs (STARS and ROSEL) comparing surgery and SBRT in operable stage I NSCLC patients were closed due to low accrual, and the two trials' pooled analysis results were limited because of the small sample size and short follow-up time (34), unfortunately. Therefore, based on the lacked evidence, we performed this meta-analysis to directly compare the effect of wedge resection and SBRT in treating early-stage NSCLC for the first time.

In our meta-analysis, six retrospective studies, with a total of 11,813 clinical stage I NSCLC patients treated with wedge resection and SBRT, were included. We chose the 5-year DFS and OS as the survival outcomes and found that wedge resection yielded a significantly better OS (HR = 1.20, 95% CI = [1.07, 1.34], *P* = 0.002) than SBRT, but there was no significant difference of DFS (HR 1.53, 95% CI = [0.83–2.83], *P* = 0.17) between wedge resection and SBRT. Our meta-analysis showed similar results with previous researches (18, 21–23). Moreover, considering potential selection bias, we also conducted subgroup analysis based on PSM, we found that survival advantages of wedge resection over SBRT still held true (HR = 1.16, 95% CI = [1.04, 1.30], *P* = 0.007). Therefore, our study proved that wedge resection had better OS than SBRT in clinical stage I NSCLC patients. In our view, three major factors contributed to the results. First, of note, surgery group innately had better physical condition than SBRT group in these retrospective studies (21). Although we strove to minimize selection bias by conducting subgroup analysis as previously described, it could not be denied that patients who underwent wedge resection generally had less underlying comorbidities than patients undergoing SBRT. For example, patients in the SBRT group were older (Table 1), and they always had more previous surgical histories, serious cerebral diseases, cardiovascular diseases, and diabetic complications (20). Second, biopsy confirmation was strongly recommended before the treatment of SBRT (35), but many patients did not receive biopsy confirmation because of the risk of developing complications. What's more, *Ajmani et al*. (22) found high-quality wedge resection (negative margins with resected lymph nodes >5) showed improved outcomes compared to lower-quality resection (positive margins or negative margins with resected lymph nodes <5). As a result, wedge resection could remove more lymph nodes, which might improve the prognosis of these patients. Therefore, high-quality wedge resection provided a more accurate pathologic diagnosis, which could guide clinicians to make proper decisions for systemic therapy (36). Third, SBRT evaluated negative preoperative staging through imaging and was lack of pathologic lymph node assessment, but wedge resection with lymph nodal sampling might remove potential metastatic lymph node that could not be recognized by pretreatment imaging, and these upstaging cases received subsequent adjuvant treatment, who might yield better LRC (18) (Table 2) and survival (37).

However, it should be noted there is still a role for SBRT in treating some specific cohorts of early-stage NSCLCs (38). By multiple planar and non-planar beams precisely targeting the tumor, SBRT has good protection of the adjacent normal tissue (7). After decades of development, SBRT technology has been proved as a standard care for early-stage NSCLC patients who are medically inoperable or unwilling to receive surgery. Clinical trial conducted by *Timmerman et al*. (10) showed inoperable NSCLC patients receiving SBRT had 3-year DFS and OS rates of 48.3 and 55.8%, respectively, and the local tumor control rate were 87.2% with moderate treatment-related morbidity. The results of this trial showed that SBRT was an excellent local therapy in medically inoperable early-stage NSCLCs. Meanwhile, in our meta-analysis, we noticed stage I NSCLC patients treated with SBRT were old people, who always had many comorbidities and poor physical condition, so that patients' follow-up could not be too long, which might explain the reason why the DFS (HR 1.53, 95% CI = [0.83–2.83], *P* = 0.17) between wedge resection and SBRT was no significantly different. Currently, many data indicated that small-size peripheral early-stage NSCLCs with low malignant characteristics had a low risk of lymph node metastasis (39), so SBRT might provide these patients a good local tumor control (10, 35). Moreover, *Peterson et al*. (40) found for tumors >5 cm, the use of SBRT resulted in excellent outcome and acceptable toxicity. As for centrally located lesions, although they had higher risk of treatment-related toxicity compared to peripheral lesions, similar dilemma still existed that centrally located tumors also could not tolerate lobectomy or sublobar resection, and therefore, SBRT is still an important choice in treating these patients (41). As a result, in our experience, SBRT is recommended to patients who decline surgery, or these who cannot tolerate surgery because of poor cardio-pulmonary function. However, the detailed criteria (age, comorbidities, physical conditions, and others) for the application of SBRT that can be useful in multidisciplinary decisions is urgently needed in clinical practice (42).

### Limitations of the Study

There are some limitations of our study. First, all the collected results were based on retrospective studies, which had low-quality evidence. Second, only two studies including 319 clinical stage I NSCLC patients provided DFS data, and the sample size was relatively small, so that we did not perform sensitivity analysis and publication bias based on DFS. Moreover, some studies didn't directly record the sufficient data, and therefore, we analyze Kaplan–Meier curves with Engauge Digitizer version 2.11, and calculated HRs with 95% CIs with spreadsheet as previously described. Third, due to the limited number of the included studies (only six sudies), the potential publication bias was observed and therefore, our meta-analysis results should be interpreted cautiously. Fourth, because 5-year OS rate of included studies ranged from 49.9 to 97.7%, which meant our meta-analysis might have high heterogeneity, although the sensitivity analysis of our meta-analysis results exhibited high stability. Finally, tumor location, tumor size and pathological subtype that might influenced long-term survival, could not be extracted from these studies to perform further subgroup analysis. Therefore, further RCTs are badly needed to confirm our conclusion.

## Conclusion

We performed the first meta-analysis to directly compare the long-term survival after wedge resection and SBRT in the treatment of clinical stage I NSCLCs. Our study notably indicated that wedge resection had better OS than SBRT in treating clinical stage I NSCLC patients, while there was no significant difference of DFS between wedge resection and SBRT. However, because there were no completed prospective RCTs, our conclusion may be interpreted with cautions before further well-designed RCTs become available.

## Data Availability Statement

The original contributions presented in the study are included in the article/Supplementary Material, further inquiries can be directed to the corresponding author/s.

## Author Contributions

LP, H-YD, Z-KL, and Q-WS collected data and drafted the manuscript. Z-KL, K-LH, Q-QZ, WL, and YW designed the study and revised the manuscript. All authors read and approved the final manuscript.

## Conflict of Interest

The authors declare that the research was conducted in the absence of any commercial or financial relationships that could be construed as a potential conflict of interest.

## Publisher's Note

All claims expressed in this article are solely those of the authors and do not necessarily represent those of their affiliated organizations, or those of the publisher, the editors and the reviewers. Any product that may be evaluated in this article, or claim that may be made by its manufacturer, is not guaranteed or endorsed by the publisher.

## Supplementary Material

Supplementary Figure 1 Sensitivity analysis for OS. CI, confidence interval; OS, overall survival.

Supplementary Figure 2 Funnel plots showed the publication bias of the analysis of OS (Egger's test, *P* = 0.003). HR, hazad ratio; OS, overall survival.
