# Supplementary material for: Wedge Resection vs. Stereotactic Body Radiation Therapy for Clinical Stage I Non-small Cell Lung Cancer: A Systematic Review and Meta-Analysis
Source: Front Surg. 2022 Mar 17;9:850276. doi: 10.3389/fsurg.2022.850276 (PMC8967956; doi:10.3389/fsurg.2022.850276)
Supplement: Supplementary Figure 1 — Sensitivity analysis for OS. CI, confidence interval; OS, overall survival. [file Table_1.DOCX]

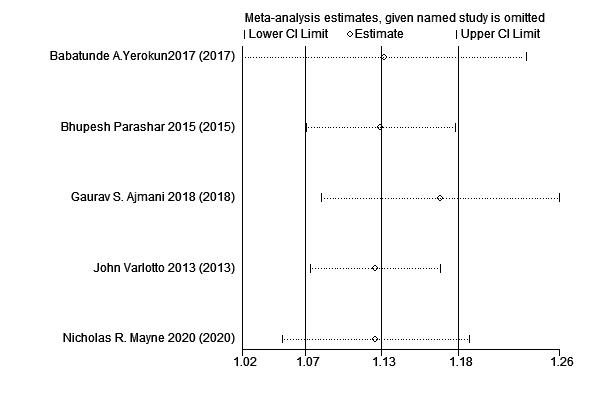


Figure 1: Sensitivity analysis for OS. CI, confidence interval; OS, overall survival.


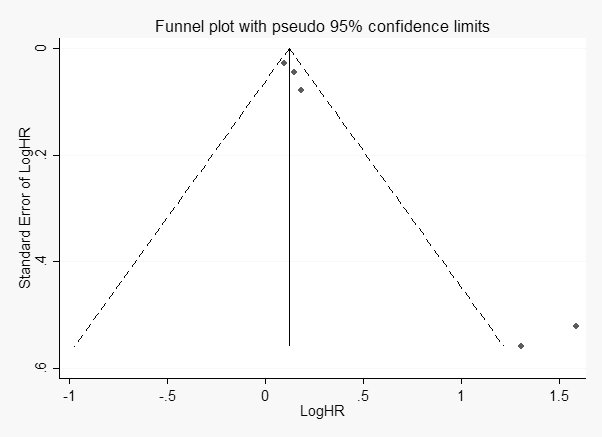


Figure 2: Funnel plots showed the publication bias of the analysis of OS (Egger’s test, P =0.003) . HR, hazad ratio; OS, overall survival.
